# Full Cross-Sectional Profile Measurement of a High-Aspect-Ratio Micro-Groove Using a Deflection Probe Measuring System

**DOI:** 10.3390/s25072335

**Published:** 2025-04-07

**Authors:** Zhong-Hao Cao, Jinyan Tang, Zhongwei Li, Yuan-Liu Chen

**Affiliations:** The State Key Lab of Fluid Power and Mechatronic Systems, Zhejiang University, Hangzhou 310027, China; caozh@zju.edu.cn (Z.-H.C.); jyttang@zju.edu.cn (J.T.)

**Keywords:** deflection measurement, high-aspect-ratio micro-groove, profile stitching, measurement system, error separation

## Abstract

**Highlights:**

**What are the main findings?**

- A deflecting-probe scanning method is proposed for the measurement of a full cross-sectional profile of high-aspect-ratio grooves.
- A method that combines a large length-to-diameter ratio probe measurement and profile stitching was used to eliminate blind areas on the groove’s side walls and bottom.

**What is the implication of the main finding?**

- The optimal deflection angle of the probe was determined based on the probe deformation and profile stitching requirements.
- A groove-scanning measurement system was developed, and a repeated scanning method was employed to accurately separate the system’s various errors.

**Abstract:**

For the full cross-sectional profile measurement of high-aspect-ratio micro-grooves, traditional measurement methods have blind measurement areas in the vertical sidewall and its intersection area with the bottom. This paper proposes a deflection-based scanning method that utilizes a large length-to-diameter ratio probe to achieve a full cross-sectional profile measurement of micro-grooves. Blind measurement areas were eliminated by a deflection-based scanning method. The complete groove profile was obtained by stitching the positive and reversal deflection-based measurement results. The optimal deflection angle of the probe was calculated by considering the profile-stitching setting and the principle of minimizing the probe deformation during the measurement process. A four-axis measurement system was established to measure high-aspect-ratio micro-grooves, which incorporated a force feedback mechanism to maintain a constant contact force during the measurement and an integrated error separation module to modify the measurement results. The measurement method and system were experimentally validated to achieve a full cross-sectional profile measurement of micro-grooves with a width of 50 μm and an aspect ratio of no less than 3. The standard deviation of the measurement results was 82 nm, and the expanded uncertainty was 108 nm.

## 1. Introduction

Micro-groove structures with a small width of tens of microns and high aspect ratios are widely used in chip manufacturing and semiconductors, including trench isolation technology, deep trench capacitors, grating structures, and microlens arrays [1,2,3,4,5,6]. In the application of precision devices, the shape accuracy and surface roughness of groove structures have a significant impact on the functionality of the devices, ultimately determining the service performance [7,8,9,10]. With the development of ultra-precision machining technology for grooves, nano-precision measurement techniques are urgently required to accurately feedback the profile information of grooves [11,12].

In recent years, extensive studies were conducted for the profile measurement of high-aspect-ratio internal features, including contact-based coordinate measurement, optical scanning imaging, and micro-CT detection [13,14,15,16]. These approaches focus primarily on the measurement of the internal sidewall of groove structures with widths in the range of 0.1 mm to 10 mm [17,18,19]. However, for the measurement of the full cross-sectional profile, including both the internal sidewall and bottom of the high-aspect-ratio grooves with a small width of less than 0.1 mm, the aforementioned approaches find it difficult to achieve accurate measurements due to some limitations [20,21,22]. Concretely, coordinate measurement methods are limited by the size of the contact probe in the tens to hundreds of microns, resulting in measurement-blind areas in the intersection area between the sidewall and the bottom [23,24]. Optical scanning imaging is a non-destructive measurement method with a high detection efficiency, but it is only suitable for the measurement of the larger-sized groove structures with a width of more than 0.1 mm [25]. Additionally, its detection capability is highly influenced by the reflectivity of the material surface [26]. Micro-CT scanning is suitable for detecting complex structures without measurement-blind areas. However, it only achieves a micron-level detection accuracy, which is insufficient for meeting the demand of nano-precision measurement [27].

Based on traditional commercial instruments, researchers proposed a series of new methods for measuring different types of grooves [28,29,30,31,32]. Yuki Shimizu et al. [33] employed a comparative measurement method on an ultra-precision grinding machine to measure the angle of a V-groove on a large ceramic workpiece with a length of 500 mm, and the approach effectively compensated for machine-induced errors in the angle measurement. X. Yin et al. [34] developed an accurate, robust, and automatic measurement system for the cross-sectional geometric parameters of various types of grooves based on machine vision detection methods. However, achieving full cross-sectional profile measurement remains challenging for grooves with a small width of tens of micros and high aspect ratios. It requires eliminating measurement-blind areas at the groove sidewalls and bottom while extracting geometric and surface parameters with nano-precision.

To achieve the measurement of a full cross-sectional profile of high-aspect-ratio micro-grooves and accurately extract shape and surface parameters, this paper proposes a deflection-based measurement method. The optimal deflection angle of the probe was determined according to a profile stitching setting and the principle of minimizing deformation. A four-axis scanning measurement system was established, and a large length-to-diameter ratio probe was developed to detect the internal profile of the high-aspect-ratio micro-grooves. The full cross-sectional profile information of high-aspect-ratio micro-grooves was obtained by stitching the positive and reversal deflection-based measurement results. The accuracy enhancement of the measurement system was achieved using an accurate error separation module. The accuracy of the proposed measurement method was validated through the calculation of the standard deviation of the measurement results and the evaluation of the measurement uncertainty.

## 2. Measurement System and Method

### 2.1. Measurement System and Method with Deflection Probe

**Figure 1:**
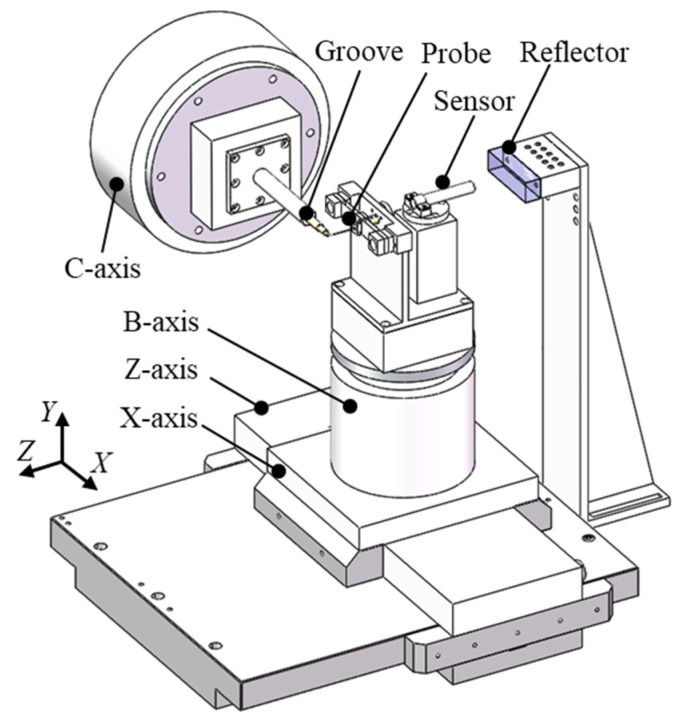
Schematic of the constructed system for the measurement of high-aspect-ratio groove.

To measure the full cross-sectional profile of the high-aspect-ratio micro-groove and eliminate the measurement blind areas at the bottom and sidewalls, a measurement system with a deflection probe was developed, as shown in Figure 1. The basic components of the system consisted of four motion axes: X, Z, B, and C. The groove was fixed on the C-axis, which rotated to change the measurement cross-section. The probe was mounted on a combination of the X-, Z-, and B-axes, with the X- and Z-axes used to calibrate the relative position between the probe and the workpiece. The B-axis adjusted the deflection angle of the probe, and the coordinated movement of the four axes enabled the measurement of the full cross-sectional profile of the groove.

To improve the measurement accuracy of the system, a sensor and a high-precision reflector were installed at the reverse position of the probe to detect linearity errors of the motion axes. The mirror was fixed on the measurement device and remained stationary, while the sensor and probe were mounted on the B-axis and moved together during the measurement process. The probe outputted the measurement results of the groove profile, and the sensor fed back the motion axis error by detecting the reflector profile variations.

**Figure 2:**
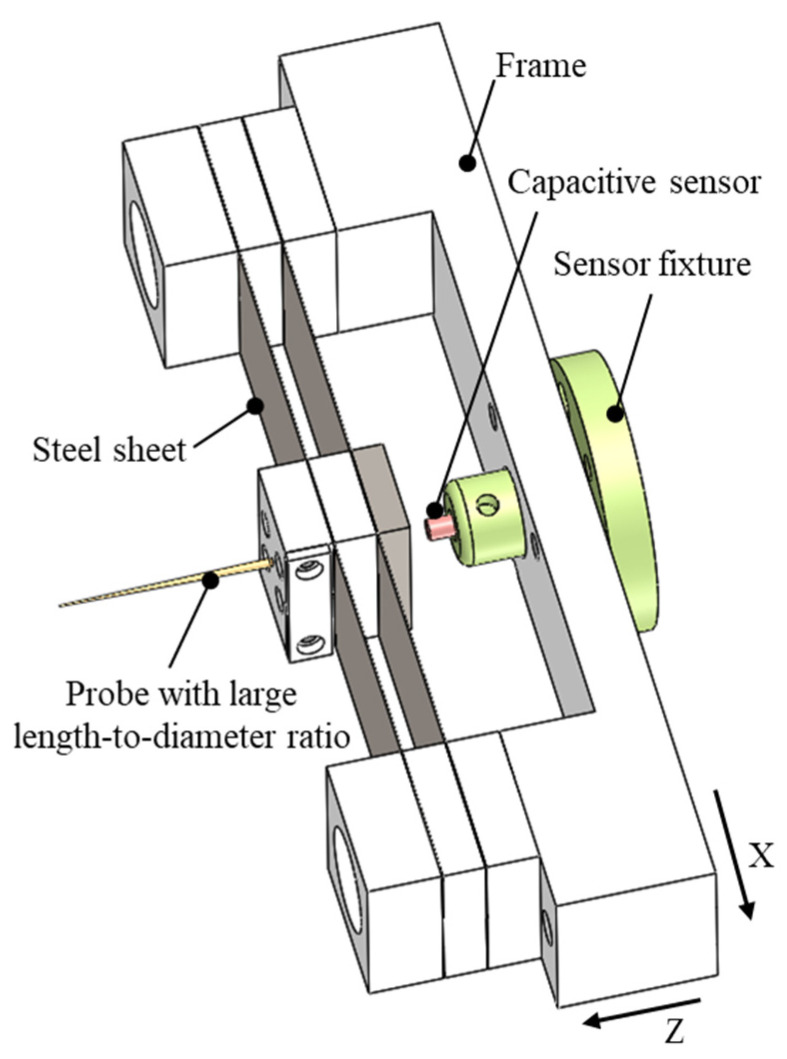
Structure of force feedback probe with large length-to-diameter ratio.

The groove exhibited a high aspect ratio, which required the probe used for the bottom profile measurement to have a large length-to-diameter ratio. The probe was mounted on a force feedback structure, as shown in Figure 2. This structure consisted of a large length-to-diameter ratio probe, dual-layer leaf springs, and a capacitive sensor. It converted changes in the workpiece profile detected by the probe tip into output signals from the capacitive sensor, which were collected and processed by the signal detection system [35,36,37].

The stiffness matrix of the force feedback structure was determined using the finite element method [38]. A force was applied along the probe’s axial direction, and the corresponding elastic deformation was calculated. Finally, the stiffness of the structure in the axial direction of the probe was obtained using Hooke’s law, expressed as the ratio of the applied force to the resulting deformation. To avoid probe wear on the surface of the groove, the stiffness of the force feedback structure was adjusted down to 1.2 mN/μm by adjusting the size of the leaf springs.

During the measurement process, the probe tip came into contact with the workpiece, and the contact force caused a deformation of the double-layer leaf springs. The capacitive sensor, which functioned as a displacement sensor, accurately detected the deformation of the leaf springs and provided feedback on the workpiece profile. To ensure accurate detection of the workpiece profile, the contact force between the probe and the workpiece needed to remain stable and constant. The force feedback structure was fixed on the X, Z, and B combined axes, and the relative position between the probe and workpiece was adjusted via the X- and Z-axes based on the detected contact force.

### 2.2. Measurement Procedure for High-Aspect-Ratio Micro-Groove

**Figure 3:**
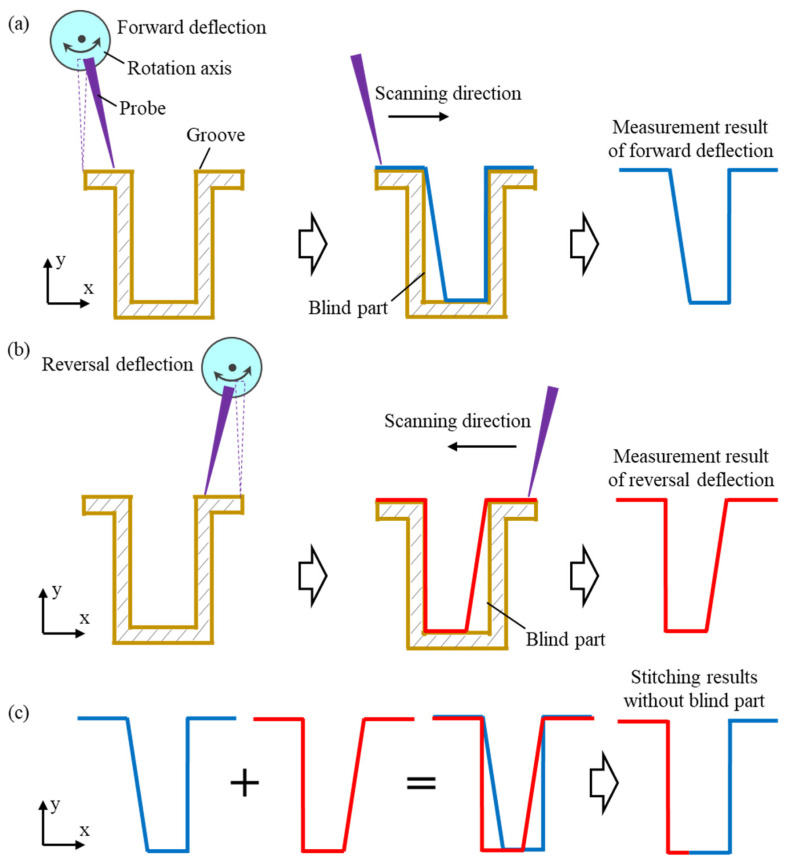
Schematic of measuring a high-aspect-ratio groove with a deflected probe. (**a**) Measurement process of forward deflection of the probe. (**b**) Measurement process of the reversal deflection of the probe. (**c**) Splicing the measurement results of the forward deflection and reversal deflection.

The deflection probe scanning and profile stitching methods were employed to measure the full cross-sectional profile of the high-aspect-ratio micro-groove. The basic measurement steps are depicted in Figure 3. The probe was mounted on a rotary axis to enable an angular deflection. The complete cross-sectional profile of the high-aspect-ratio micro-groove was reconstructed by stitching the profiles obtained from two deflected probe measurements. As shown in Figure 3a, the probe was deflected forward and scanned along the groove profile in the forward direction during the measuring process. Partial groove profile information was acquired by extracting the position information of the probe during scanning. After the forward measurement was completed, as depicted in Figure 3b, the probe was reverse-deflected through the rotation axis and scanned along the groove in the reverse direction. Then, the profile information of the other side of the groove was obtained through the output of the probe coordinates. Each of the two measurements contained blind areas at different locations and overlapping regions in the measured profiles. To eliminate the blind areas, the complete cross-sectional profile of the groove was obtained by stitching the measurement results based on the overlapping regions of the two measured profiles, as shown in Figure 3c.

To measure the profile of the groove sidewalls, the probe was deflected at a specific angle to ensure a stable contact between the probe tip and the sidewall. However, the deflected probe created blind areas at the intersection of the sidewall and bottom surfaces. These blind areas could be eliminated by conducting measurements with the probe deflected in two directions. Overlapping regions were present in the measurements of the groove bottom profile. By stitching these overlapping regions, the two measurement results complemented each other, which effectively eliminated the blind areas from the individual measurements and enabled the detection of the complete cross-sectional profile of the groove.

During the scanning measurement along the groove profile, a force feedback mechanism controlled the contact force between the probe tip and the groove surface. The relative position of the probe and the groove was adjusted using a force feedback method to ensure a constant contact force throughout the measurement process. After the measurement was completed, the groove profile was reconstructed based on the coordinate data obtained from the probe.

**Figure 4:**
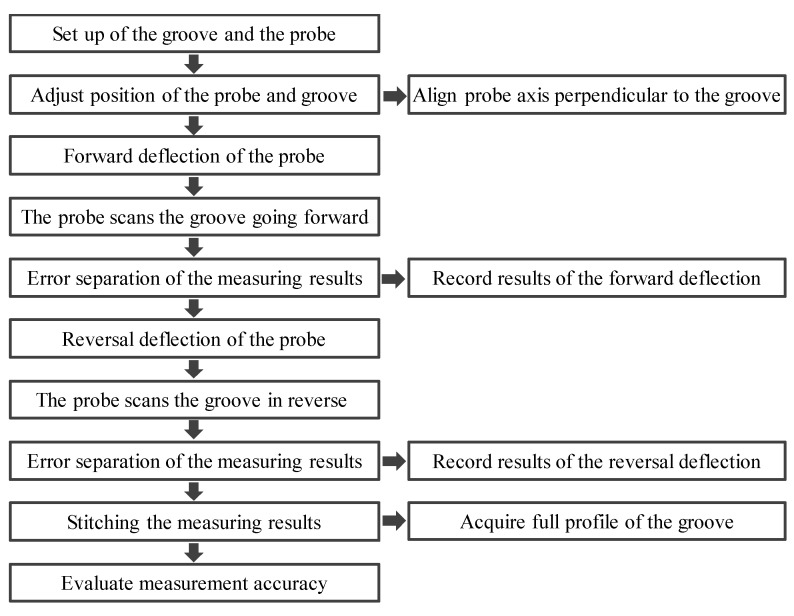
Schematic of the measurement process of the high-aspect-ratio groove profile.

The process for measuring the complete cross-sectional profile of the high-aspect-ratio micro-groove is illustrated in Figure 4. The local profile of the groove was detected using the deflected probe scanning method. After the forward deflection measurement was completed, the probe was deflected in the reverse direction to repeat the measurement. The complete cross-sectional profile of the groove was then obtained by stitching the results from the two measurements. The relative position between the probe and the workpiece was calibrated before the measurement to enhance the measurement accuracy. Measurement system errors were monitored during the process, and measurement errors were compensated for and eliminated in the results. The reliability was further improved by calculating the standard deviation and measurement uncertainty through repeated measurements.

## 3. Measurement Experiments

### 3.1. Analysis and Calculation of the Deflection Angle

**Figure 5:**
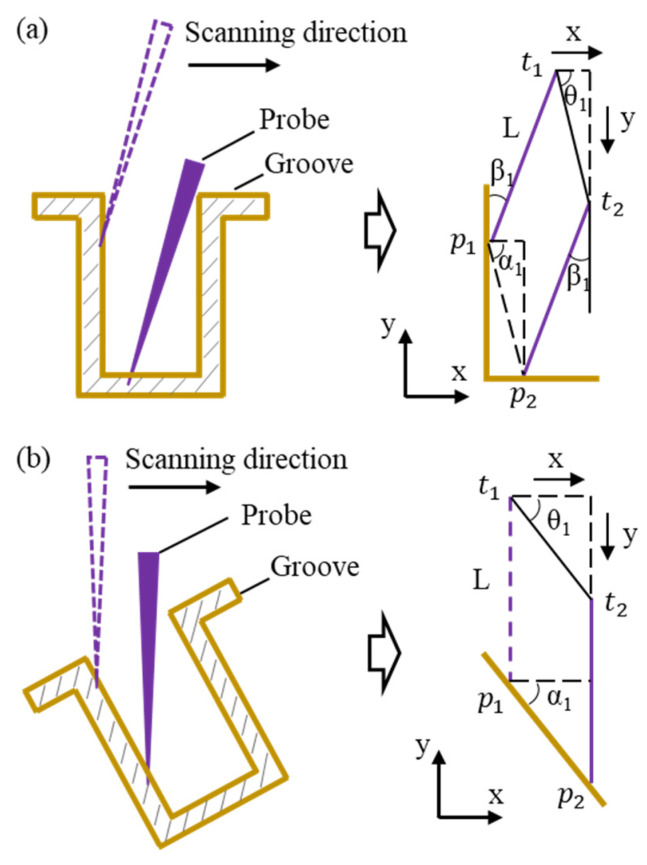
The model of the influence of the deflection angle on the accuracy of the measurement results. (**a**) Deflection of the probe. (**b**) Deflection of the groove.

To detect the sidewalls of the high-aspect-ratio micro-grooves effectively, the probe and workpiece were deflected at specific angles during the measurement process. As shown in Figure 5, the effects of the probe deflection and workpiece deflection on the accuracy of the measurement results were analyzed. In the case of a deflected probe, as illustrated in Figure 5a, the probe scanned along the detection direction, and the variation of the profile position was *t*_1_*t*_2_ based on the probe coordinates. The variation formed an angle *θ*_1_ with the X-axis. The actual profile variation was *p*_1_*p*_2_, which formed an angle *α_1_* with the X-axis. By setting point *p_1_* as the coordinate origin, the coordinates of each point were defined as *p*_1_ (0, 0), *p*_2_ (*x*, *−y*), *t*_1_ (L*sinβ_1_, L*cosβ_1_), and *t*_2_ (L*sinβ_1_ *+ x*, L*cosβ_1_ *− y*), where *L* represents the probe length and *β*_1_ represents the angle between the probe and the Y-axis. The measured profile information and the actual profile information of the workpiece were described by Equations (1) and (2):

(1) $$t_{1}t_{2}=p_{1}p_{2}=\sqrt{x^{2}+y^{2}}$$

(2) $$\alpha_{1}=\theta_{1}=\tan^{-1}\left(y/x\right)$$

Among them, *x* represents the distance the probe moved along the groove profile in the X-direction, and *y* represents the distance the probe moved in the Y-direction. From Equations (1) and (2), it was determined that the probe measurement results were consistent with the actual profile variation of the workpiece. This demonstrated that the deflected probe measurement method effectively detected the workpiece profile.

For the case of a deflected workpiece with an unchanged probe, as shown in Figure 5b, the calculation method remained the same as in Equations (1) and (2). The measurement results were consistent with the actual profile of the workpiece. This indicated that in the deflection-based measurement method, the workpiece profile information could accurately measure whether the probe or the workpiece was deflected.

**Figure 6:**
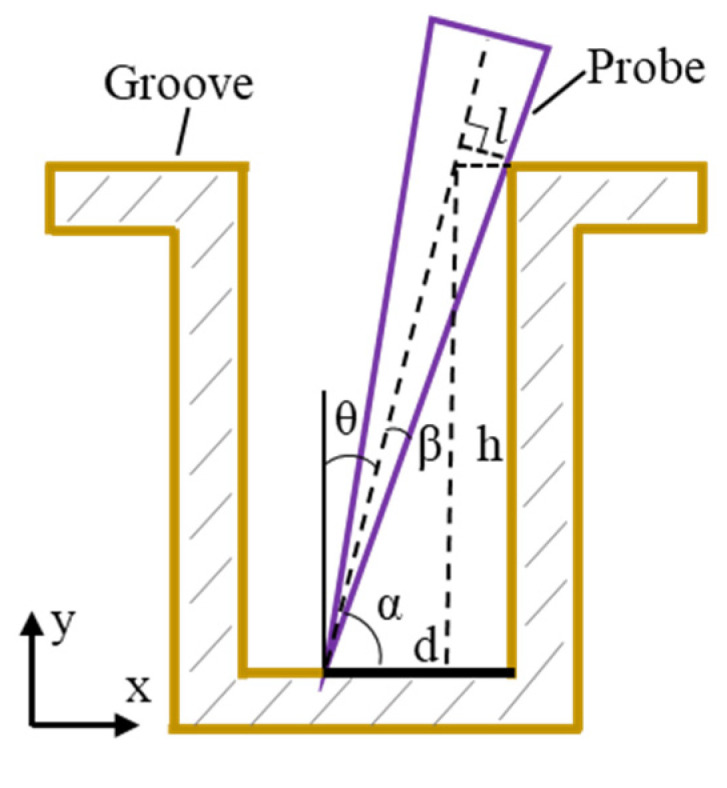
Calculation model of maximum deflection angle to avoid interference between probe and groove during measurement.

The deflection-based measurement method obtained the full cross-sectional profile of the workpiece by stitching the results of two probe scans. The stitching process utilized the overlapping region at the bottom of the workpiece, which required the range of a single measurement to exceed half of the bottom region. The probe’s deflection angle range was constrained based on this measurement requirement. A probe deflection measurement model was established, as shown in Figure 6, and the formula for calculating the maximum deflection angle *θ* was derived as follows:

(3) $$l=\sqrt{d^{2}+h^{2}}\times \sin\beta ,\;\beta =\tan^{-1}\left(1/2n\right)$$

(4) d×sin⁡α−h×cos⁡α=l

(5) $$\sin\theta =\cos\alpha =\left(-2lh+\sqrt{{\left(2lh\right)}^{2}-4\left(h^{2}+d^{2}\right)\left(l^{2}-d^{2}\right)}\right)/\left(2\times \left(h^{2}+d^{2}\right)\right)$$

where *l* represents the distance from the probe’s contact point with the workpiece to the probe axis, *β* denotes the angle between the probe axis and the generatrix, *α* refers to the angle between the probe axis and the bottom surface of the workpiece, *h* indicates the groove depth, *d* is the distance from the probe tip to the groove sidewall, *n* represents the probe’s length-to-diameter ratio, and *k* denotes the groove’s depth-to-width ratio.

To meet the requirements for stitching the bottom profile of the workpiece, the range of a single measurement needed to exceed half of the bottom profile. The measurement regions overlapped, which satisfied the following formula:

(6) d<h/2k

The maximum deflection angle *θ* of the probe was

(7) $$\theta =\sin^{-1}\left(2\left(n-1\right)/\left(\sqrt{4n^{2}+1}\times \sqrt{4k^{2}+1}\right)\right)$$

Equation (7) shows that to ensure the probe’s single measurement range exceeded half of the workpiece’s bottom profile, the probe’s deflection angle depended only on the probe’s length-to-diameter ratio *n* and the groove’s depth-to-width ratio *k*. When *n* = 10 and *k* = 3, the maximum deflection angle of the probe was 8.5°.

**Figure 7:**
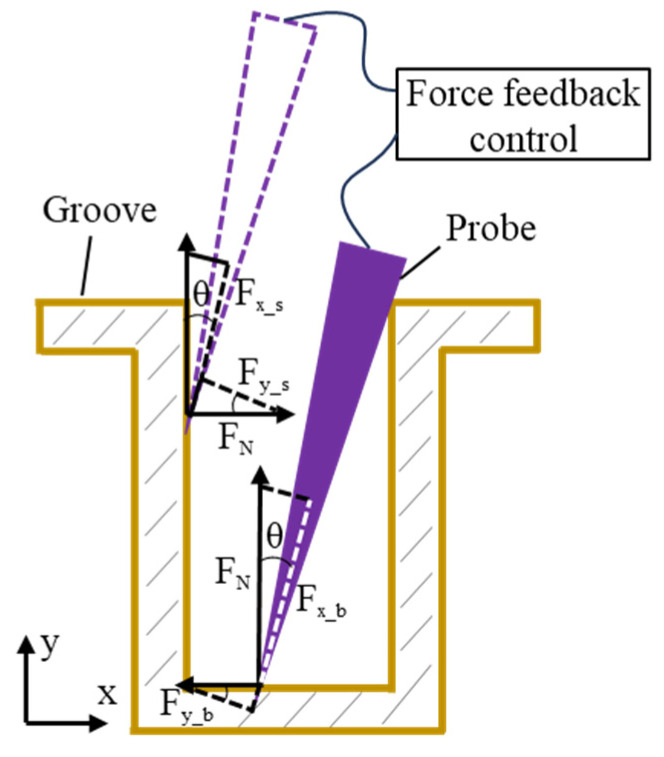
Force analysis of the probe contact with the groove sidewall and bottom.

As a contact-based measurement method, probe scanning introduced deformation when the probe tip contacted the groove’s sidewall and bottom surface, which led to measurement errors. To minimize the deformation at the probe’s tip, the contact force state between the probe tip and the workpiece was analyzed, as shown in Figure 7. The forces exerted on the probe during contact with the groove’s sidewall were calculated, including the force along the probe’s axial direction *Fx_s* and the force perpendicular to the probe’s axial direction *Fy_s*:

(8) $$F_{x\_s}=F_{N}\left(\sin\theta +k\cos\theta \right)$$

(9) $$F_{y\_s}=F_{x\_s}\times \left(1-k\tan\theta \right)/\left(\tan\theta +k\right)$$

where *k_f_* represents the coefficient of friction between the probe and the workpiece surface, *F_N_* denotes the support force from the workpiece surface, and *θ* refers to the angle between the probe axis and the vertical direction.

Similarly, when the probe contacted the bottom profile of the workpiece, the forces acting on the probe were derived. The force along the probe’s axial direction *Fx_b* and the force perpendicular to the probe’s axial direction *Fy_b* were calculated as follows:

(10) $$F_{x\_b}=F_{N}\left(\cos\theta -k\sin\theta \right)$$

(11) $$F_{y\_b}=F_{x\_b}\times \left(\tan\theta +k\right)/\left(1-k\tan\theta \right)$$

The probe tip was mounted on the force feedback structure shown in Figure 4. This structure adjusted the force along the probe’s axial direction through feedback regulation, which ensured that *Fx_s* and *Fx_b* remained constant during the measurement process. The overall stiffness of the force feedback structure was 1.2 mN/μm, and the deformation of the leaf spring during the measurement process was controlled at 0.4 μm. Consequently, the contact force *Fx_s* and *Fx_b* between the probe and the workpiece along the probe’s axial direction was calculated to be 0.48 mN. Furthermore, based on Equations (9) and (11), it was determined that to reduce the deformation at the probe tip caused by the applied forces, it was only necessary to adjust the probe’s deflection angle to minimize the force perpendicular to the probe axis and the deformation caused by the axial forces.

**Figure 8:**
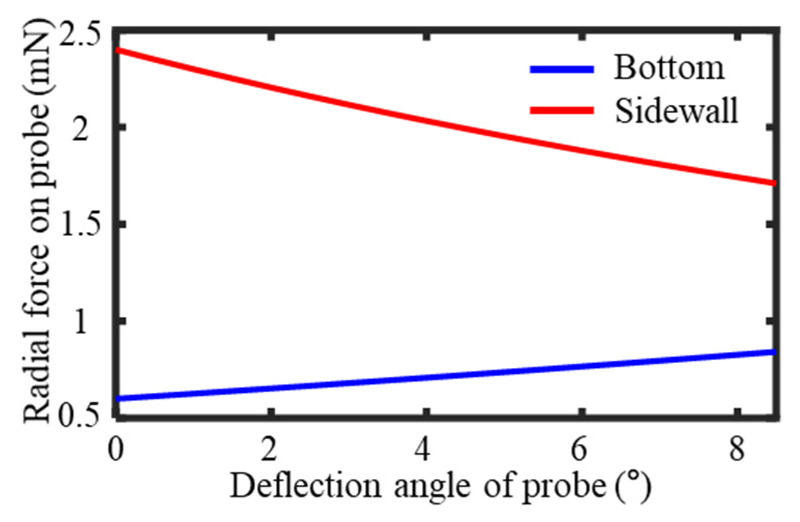
Radial force of probe in contact with groove at different deflection angles.

According to the above analysis, it was determined that the probe’s deflection angle range needed to be 0–8.5° to meet the profile stitching requirements for a single measurement. Within this deflection angle range, the force acting perpendicular to the probe’s axis was calculated based on the deflection angle using Equations (9) and (11), as shown in Figure 8. As the probe’s deflection angle increased, the contact force between the probe and the sidewall gradually decreased, while the contact force with the bottom surface continuously increased.

**Figure 9:**
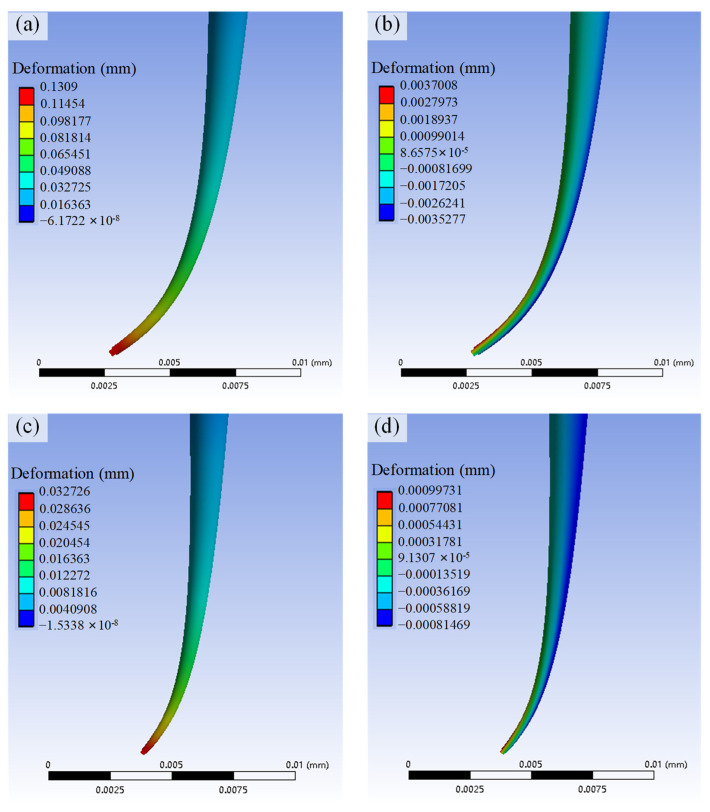
Simulation analysis of the deformation of a probe under a contact force. (**a**) Radial deformation of the probe in contact with the sidewall of the groove. (**b**) Axial deformation of the probe in contact with the sidewall of the groove. (**c**) Radial deformation of the probe in contact with the bottom of the groove. (**d**) Axial deformation of the probe in contact with the bottom of the groove.

The deformation of the probe was simulated based on the force acting on the probe tip, as shown in Figure 9a–d. The axial and radial deformations of the probe during contact with the groove’s sidewall and bottom surface were calculated for different deflection angles. Due to the probe’s high length-to-diameter ratio, the deformation was primarily concentrated at the probe tip.

**Figure 10:**
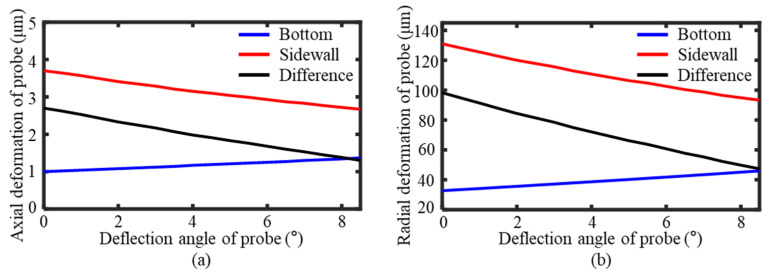
Deformation of probe in contact with groove at different deflection angles. (**a**) Axial deformation. (**b**) Radial deformation.

To enhance the stability of the measurement process and minimize the errors, the deformation of the probe needed to remain consistent. It required minimizing the difference between the axial and radial deformation values when the probe contacted the sidewall and bottom surface of the groove. The difference in the axial deformation under varying deflection angles was calculated based on force contact simulations, as shown in Figure 10a. As the deflection angle increased, the difference in the axial deformation between the sidewall and the bottom surface contact decreased. Similarly, as shown in Figure 10b, the difference in the radial deformation also reduced with increasing deflection angles. Therefore, larger deflection angles resulted in a greater measurement stability within the deflection range of 0–8.5°.

Considering the overlap area of the measurement results and the probe deformation, the probe deflection angle was set to 7° during the measurement process. At this deflection angle, the difference in the axial deformation between the probe’s contact with the sidewall and the bottom surface was 1.55 μm, while the difference in the radial deformation was 56.5 μm. The probe exhibited stable deformation during the measurement process, which allowed the deformation values to be directly compensated in the measurement results, which eliminated the measurement errors caused by the probe tip deformation.

### 3.2. Experimental Setup

**Figure 11:**
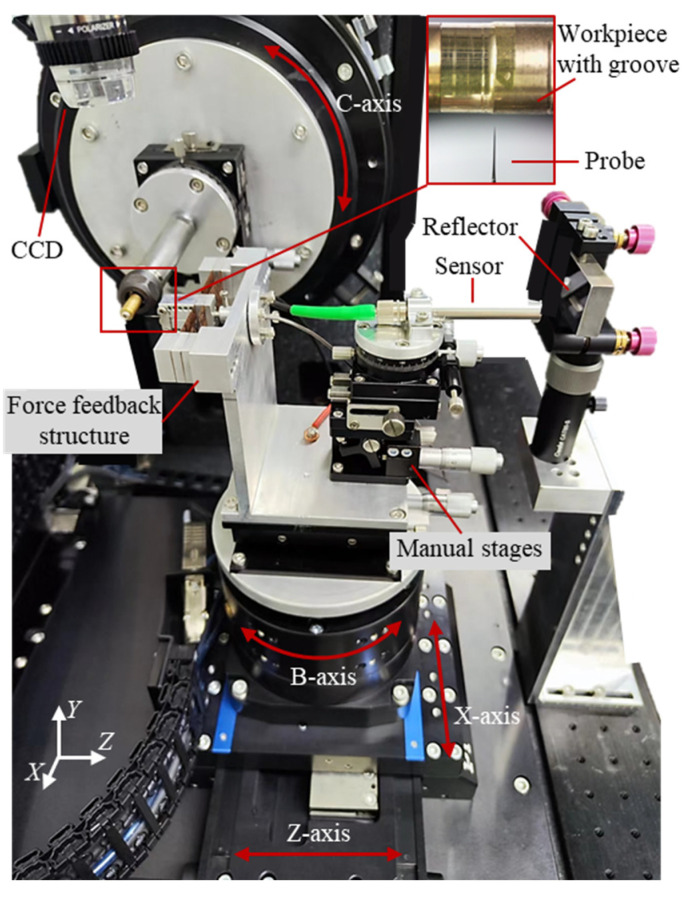
Experimental device for measuring groove profile by deflection probe.

To achieve a full cross-sectional profile measurement of the high-aspect-ratio micro-grooves, a probe scanning measurement device was developed based on the deflected probe-scanning method, as shown in Figure 11. The workpiece with groove features was clamped on the C-axis using a collet chuck. The force feedback structure, which held the probe, was mounted on the X-, Z-, and B-axis combination using a manual displacement stage. This configuration allowed for the adjustment of the relative position between the workpiece and the probe by combining manual displacement stages with motorized axes. The laser sensor was positioned using a manual adjustment stage, which enabled it to measure the fixed reflector on the equipment and output the linearity error of the motion axis. Two sets of CCD sensors arranged orthogonally in the measuring device were used to monitor the measurement position of the probe. During the measurement, the motion axis drove the probe to detect the workpiece profile. The force feedback structure, laser sensor, and motion axes synchronized their outputs, which were subsequently used to reconstruct the groove profile.

**Figure 12:**
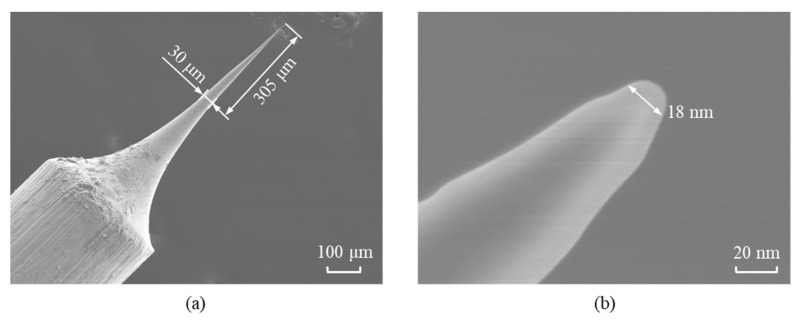
Tungsten probe processed by the electrochemical corrosion method. (**a**) Overall profile of the probe with large length-to-diameter ratio. (**b**) Probe tip.

The groove to be measured had a width of approximately 50 μm and a depth of about 150 μm, which resulted in a depth-to-width ratio of approximately 3:1. The probe used for the detection required a length-to-diameter ratio significantly greater than the groove’s depth-to-width ratio, where the tip diameter determined the lateral resolution of the scanning measurement. A high-hardness tungsten wire was used as the raw material to meet the experimental requirements, and a high-aspect-ratio probe was fabricated through an electrochemical etching process. The dimensions of the probe are shown in Figure 12, with a length-to-diameter ratio of approximately 10:1 and a tip diameter of 18 nm, fulfilling the requirements for profiling high depth-to-width ratio grooves.

To improve the measurement accuracy, the primary components of the measurement system were customized, including the X and Z linear axes, the capacitive sensor within the force feedback structure, and the laser sensor used for detecting the linearity errors. The specific parameters of the measuring device are listed in Table 1.

### 3.3. Calibration of the Measuring Probe

**Figure 13:**
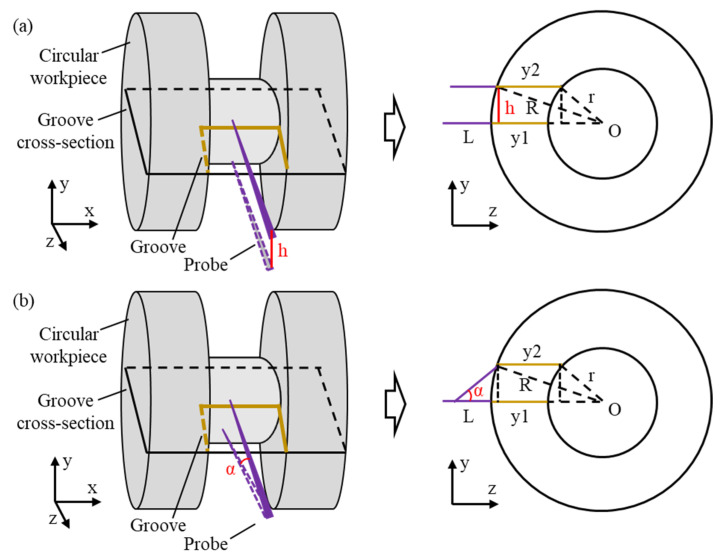
The measurement error model caused by the position deviation of the probe relative to the groove section. (**a**) The probe with a deviation distance of h. (**b**) The probe with a deviation angle of α.

The measurement error of the groove profile consisted of the positional deviation of the probe relative to the workpiece before measurement and the motion axis error during the measurement process. The motion axis error was detected using the integrated laser sensor within the measurement system and subsequently separated from the measurement results. The positional deviation of the probe relative to the workpiece was corrected before measurement. As shown in Figure 13, the groove structure was distributed on a circular workpiece, with the positional deviation of the probe primarily caused by the deviation distance along the Y-axis and the deviation angle around the X-axis.

As shown in Figure 13a, the top radius of the annular groove was *R*, the full length of the probe was *L*, and the bottom diameter was *r*. In the absence of error, the probe axis was perpendicular to the cross-section of the groove at its maximum diameter. When the probe deviated by a distance *h* along the Y-axis, the resulting measurement error *E*_1_ was

(12) $$E_{1}=\sqrt{R^{2}-h^{2}}-\sqrt{r^{2}-h^{2}}-R+r$$

When the probe rotated around the X-axis by an angle *α*, as shown in Figure 13b, the resulting measurement deviation *E*_2_ was

(13) $$E_{2}=\sqrt{R^{2}-{L\sin\alpha }^{2}}-\sqrt{r^{2}-{L\sin\alpha }^{2}}-R+r$$

**Figure 14:**
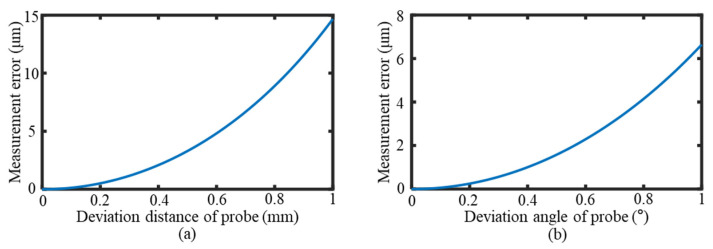
The measurement error caused by the position deviation of the probe relative to the groove section. (**a**) The measurement error caused by the deviation distance. (**b**) The measurement error caused by the deviation angle.

From Equations (12) and (13), it was concluded that the measurement error increased continuously with the probe deviation distance *h* and the deviation angle *α*. The variation trends are shown in Figure 14a,b. The measurement errors caused by the probe deviation distance and deviation angle were significant and required a correction of the probe position before measurement to eliminate the positional deviations.

**Figure 15:**
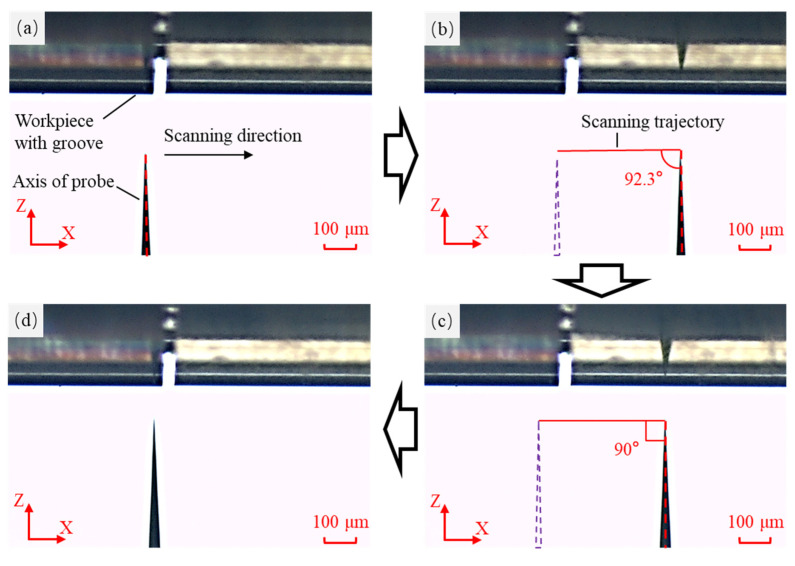
The position adjustment process of the probe in the X-Z plane. (**a**) The initial position of the probe. (**b**) The position of the probe after moving along the X-direction. (**c**) The probe axis was adjusted perpendicular to the trajectory. (**d**) The probe was moved to the initial measurement position.

To determine the actual deflection angle of the probe, the probe axis was adjusted to be perpendicular to the scanning trajectory before the deflection, with the deflection angle set to 0°. Under the monitoring of the CCD sensors, the initial state of the probe was recorded, as shown in Figure 15a, which documents the direction of the probe axis. The probe was then scanned along the X-axis for a certain distance, as shown in Figure 15b, and the angle between the probe axis and the scanning trajectory was measured. The probe axis angle was adjusted by the manual stage so that the probe axis was perpendicular to the scanning trajectory, which reduced the angle from 92.3° to 90°, as illustrated in Figure 15c. After the angle adjustment, the probe was moved back to its initial measurement position, as depicted in Figure 15d. These steps completed the position adjustment of the probe in the X-Z plane.

**Figure 16:**
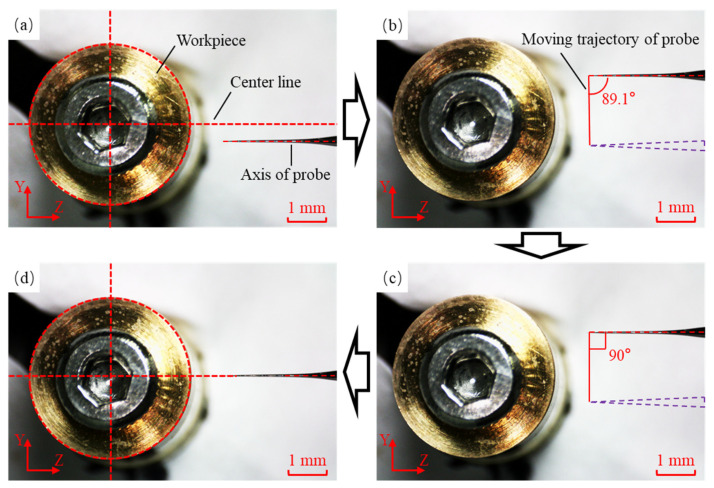
The position adjustment process of the probe in the Y-Z plane. (**a**) The initial position of the probe. (**b**) The position of the probe after moving along the Y-direction. (**c**) The probe axis was adjusted perpendicular to the trajectory. (**d**) The probe was moved to the maximum diameter of the workpiece.

In the Y-Z plane, the probe tilt angle was adjusted to ensure the probe was installed horizontally, with its axis aligned with the maximum diameter direction of the workpiece cross-section. The initial position of the probe in the Y-Z plane is shown in Figure 16a, where the probe exhibited a deviation angle and deviation distance relative to the workpiece. The probe was scanned along the Y-axis, as shown in Figure 16b, and the angle between the probe axis and the scanning trajectory was recorded. The probe position was adjusted to change the angle from 89.1° to 90° by a manual stage, as depicted in Figure 16c. After the angle adjustment process was completed, the probe axis became perpendicular to the Y-axis. The probe was then moved to the maximum diameter of the workpiece cross-section, as shown in Figure 16d. Through the above steps, the probe position adjustment process was completed.

### 3.4. Experimental Process of the Deflection Measurement Method

**Figure 17:**
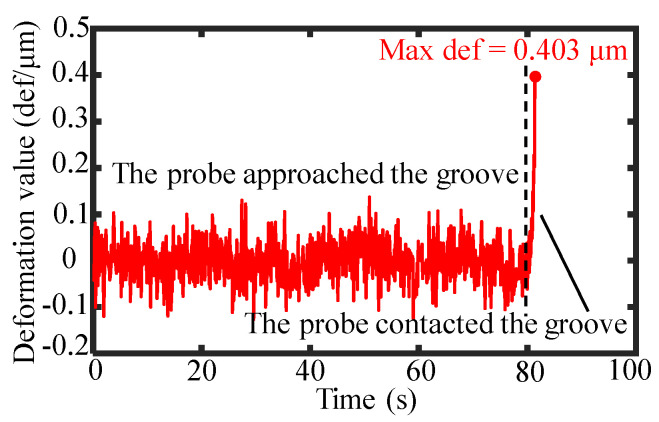
The deformation of the leaf spring during the contact process between the probe and the workpiece with the groove.

Based on the constructed measurement system, a deflection-type probe with a high length-to-diameter ratio was employed to measure the profiles of the grooves with a high depth-to-width ratio. The probe was mounted on the force feedback structure and gradually approached the workpiece surface, and the capacitive sensor within the force feedback structure synchronously outputted the contact status signal, as shown in Figure 17. The static noise of the capacitive sensor was ±0.1 μm. When the probe came into contact with the workpiece, the output of the capacitive sensor increased significantly. The force feedback structure set the capacitive sensor output to 0.4 μm when the probe was in stable contact with the workpiece. Given the stiffness of the force feedback structure was 1.2 mN/μm, the contact force between the probe and the workpiece was calculated as 0.48 mN. Maintaining a stable probe contact force of around 0.48 mN during the measurement through a force feedback structure could effectively avoid noise interference from the capacitive sensors. A low contact force could prevent damage to the workpiece.

**Figure 18:**
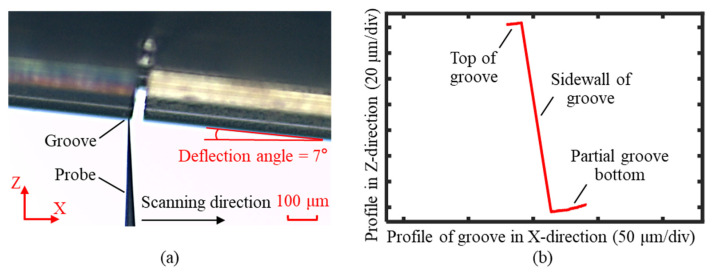
Measurement process of the groove with a forward deflection probe. (**a**) The measurement position of the probe relative to the groove, with a deflection angle of 7°. (**b**) Original measurement results of the groove profile with a forward deflection probe.

The deflection probe measurement process is illustrated in Figure 18a. The probe axis was aligned relative to the workpiece by rotating the B-axis, with the deflection angle set to 7°. Under the control of the force feedback structure, the probe scanned along the X-axis at a constant force. The X-axis and Z-axis coordinates were simultaneously recorded and used to reconstruct the groove profile, as shown in Figure 18b. Due to the deflection angle during the measurement, the reconstructed groove profile exhibited an overall tilt. The groove width was approximately 50 μm, and to satisfy the profile stitching requirements, the measured range at the groove’s bottom exceeded 25 μm.

**Figure 19:**
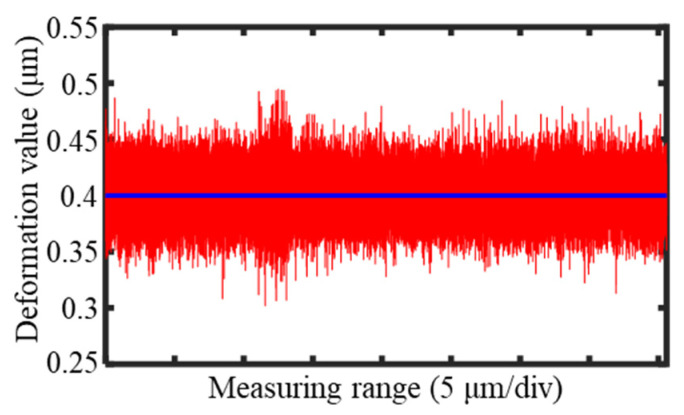
The deformation of the leaf spring during the measurement of the groove.

The force feedback structure that held the probe used a capacitive sensor to detect the deformation of the leaf spring, which provided feedback on the workpiece’s profile. During the measurement process, the capacitive sensor continuously outputted data, as shown in Figure 19. The sensor output remained stable at approximately 0.4 μm, with an error of ±0.05 μm, which aligned closely with the system’s predefined value and verified the system’s stability. When measuring the groove sidewalls, the steep inclination of the sidewalls caused fluctuations in the sensor output, which increased the error signal to ±0.1 μm. However, the average output value remained at 0.4 μm, with the error within an acceptable range, which ensured that the groove profile measurement accuracy was unaffected.

When the probe made contact with the surface of the workpiece, the output of the sensor was approximately 0.4 μm, which was significantly greater than the noise interference signal. During the measurement of the entire cross-section of the groove, if the output of the sensor remained around 0.4 μm, this proved that the probe was constantly in contact with the surface of the workpiece, and the output of the sensor was caused by the deformation of the leaf spring that resulted from the force applied to the probe. According to the output results of the sensor shown in Figure 19, it could be concluded that the probe maintained continuous contact with the workpiece during the scanning process, and the contact force was stable.

In the measurement experiment, the single scanning range of the probe was 28 μm, the scanning speed was 0.05 μm per second, and the single measurement time was 9.3 min. The steps of the forward deflection scan and the reverse deflection scan of the probe were the same. The total measurement time for the two scans was 18.6 min. Taking into account the time for the probe to approach the surface of the workpiece for initialization and the time for the probe to return to the measurement zero point, the measurement time for the groove cross-section with a cross-section length of approximately 50 μm was about 20 min.

In the measurement device we built, the probe was made of tungsten with high hardness, which features good wear resistance. During the scanning time of a single groove, the wear of the probe was negligible. To facilitate the machining of high-aspect-ratio groove structures on the workpiece, copper was chosen as the material for the workpiece. Since the contact force between the probe and the workpiece was 0.48 millinewtons, the probe did not cause obvious wear on the surface of the workpiece.

The measurement result *O__probe_*, which was obtained by scanning the workpiece profile with the probe, comprised the actual groove profile *P__groove_* and the X-axis straightness error *E__straightness_*, as described by Equation (14). The motion axis straightness error needed to be separated from the measurement results to enhance the system’s measurement accuracy.

(14) $$O_{\_probe}=P_{\_groove}+E_{\_straightness}$$

In the measurement setup shown in Figure 11, a laser sensor was integrated along the same axis as the probe. The laser sensor detected a reflector fixed to the equipment to provide feedback on the X-axis straightness error. The laser sensor and probe simultaneously generated output signals during the measurement process. The laser sensor’s output *O__sensor_*_1_ contained information about the reflector profile *P__reflector_*, the mirror installation error *E__installation_*, and the probe output *O__probe_*, as described in Equation (15). The mirror installation error, categorized as a linear error, was eliminated through data fitting.

(15) $$O_{\_sensor1}=P_{\_reflector}+E_{\_installation}+O_{\_probe}$$

After completing the measurement, the force feedback structure was deactivated, and the laser sensor performed a straight-line scan along the measurement direction. The second output *O__sensor_*_2_ from the laser sensor included the reflector profile *P__reflector_*, the mirror installation error *E__installation_*, and the X-axis straightness error *E__straightness_*, as shown in Equation (16):

(16) $$O_{\_sensor2}=P_{\_reflector}+E_{\_installation}+E_{\_straightness}$$

**Figure 20:**
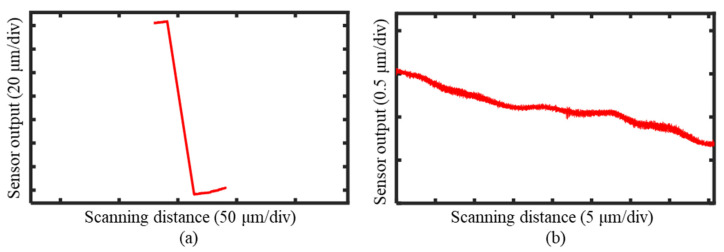
The measurement results of the laser sensor were used to separate the measurement errors. (**a**) The measurement results of the laser sensor following the probe scanning. (**b**) The measurement results of laser sensor scanning in a straight line along the X-direction.

**Figure 21:**
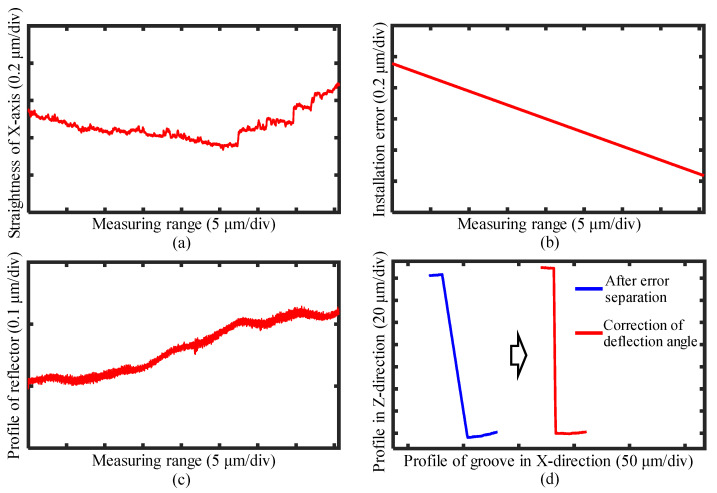
Error separation of the measurement results for the groove. (**a**) Straightness of the X-axis within the measuring range. (**b**) Installation error of the reflector. (**c**) Profile of the reflector within the measuring range. (**d**) Profile of the groove after the error separation and angle correction.

The measurement results obtained by the laser sensor during the probe scanning process are shown in Figure 20a, while the results from the linear scanning along the X-axis are presented in Figure 20b. By combining Equations (14)–(16), all the measurement system errors were successfully separated. The straightness error of the X-axis within the measurement range is illustrated in Figure 21a. The installation error of the reflector separated by the linear fitting is shown in Figure 21b, and the reflector profile information is depicted in Figure 21c. After separating these errors from the groove profile measurement results, the tilt angle of the workpiece relative to the scanning probe was corrected based on the pre-set tilt angle. The corrected groove profile is presented in Figure 21d.

The profile of the high-aspect-ratio micro-groove was measured using a deflection-based probe scanning method, which integrated a laser sensor to separate linear axis errors and achieve high-precision results. To eliminate measurement-blind areas and obtain the full cross-sectional profile of the groove, the probe was deflected in the opposite direction. The same measurement procedure was applied to obtain the profile of the other side of the groove, and the two measurement results were subsequently stitched.

**Figure 22:**
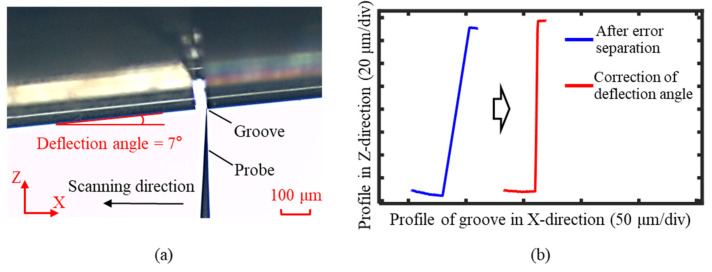
Measurement process of the groove with a reversal deflection probe. (**a**) The measurement position of the probe relative to the groove, with a deflection angle of 7°. (**b**) Measurement results with a reversal deflection probe after the error separation and angle correction.

As shown in Figure 22a, after completing the measurement with the probe in its forward deflection position, the probe was reversed to a deflection angle of −7°. The probe was then positioned at the starting point on the right side of the workpiece and scanned in the negative X-axis direction. The measurement procedure was identical to that used for the forward deflection of the probe. After measuring the groove profile, the deflection angle was corrected, and the measurement errors were separated, as illustrated in Figure 22b, which yielded the profile information for the right side of the high-aspect-ratio micro-groove. Through the aforementioned measurement steps, the profiles of both sides of the groove were successfully measured.

## 4. Discussion of the Measurement Results

### 4.1. Stitching of the Measured Profiles

**Figure 23:**
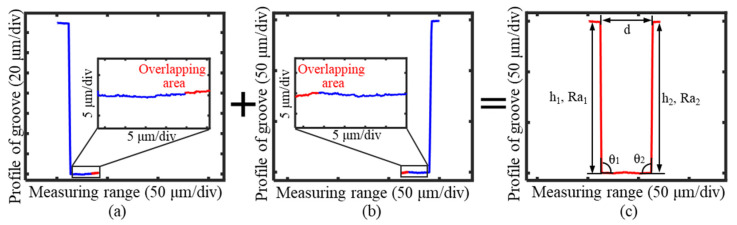
Measurement and stitching results of the groove profile. (**a**) The measurement results of the left profile of the groove. (**b**) The measurement results of the right profile of the groove. (**c**) The splicing results of the full cross-sectional profile of the groove.

To effectively extract the complete profile information of the groove, after completing the measurements of both the left and right profiles of the groove, the two results were merged to obtain the complete cross-sectional profile. The overlapping region of the two profile measurement results was at the bottom of the groove, with a range of approximately 3 μm, as shown in Figure 23a,b. The point cloud stitching method utilized the least-squares method to minimize the distances between all the overlapping points. The overlapping region at the groove bottom was successfully stitched through multiple iterative calculations, which resulted in a complete cross-sectional profile of the high-aspect-ratio micro-groove, as shown in Figure 23c.

Key parameters were extracted based on the measured groove profile to evaluate the workpiece’s surface quality. As shown in Table 2, the extracted parameters included the groove width *d*, groove depth *h*, sidewall roughness *Ra*, and the angle *θ* between the sidewall and the groove bottom.

The machining parameters for the groove were specified as a width of 50 μm and a depth of 150 μm. Based on the stitched profile data, the top width of the measured groove was 50.7 μm, while the depths of the left and right sides were 149.23 μm and 150.43 μm, respectively. The discrepancies between the measured results and the machining parameters could be attributed to machining or measurement errors. The measurement accuracy was further evaluated through the repeatability and uncertainty of the measurement results.

The measured depth-to-width ratio of the groove was approximately 3. This validated the capability of the large length-to-diameter ratio probe to measure the profiles of grooves with high depth-to-width ratios. The angles between the groove sidewalls and the bottom surface were determined to be 90.3° and 89.7°, respectively, which confirmed that the deflection probe measurement method effectively eliminated blind areas and accurately captured the interfacial angle information.

Additionally, the roughness values of the sidewall surface were calculated to be 55 nm and 58 nm, which reflected the machining precision of the groove profile. These results demonstrate the ability of the measurement system to provide accurate and detailed evaluations of the groove’s geometric and surface parameters.

### 4.2. Calculation of the Measurement Standard Deviation and Uncertainty

**Figure 24:**
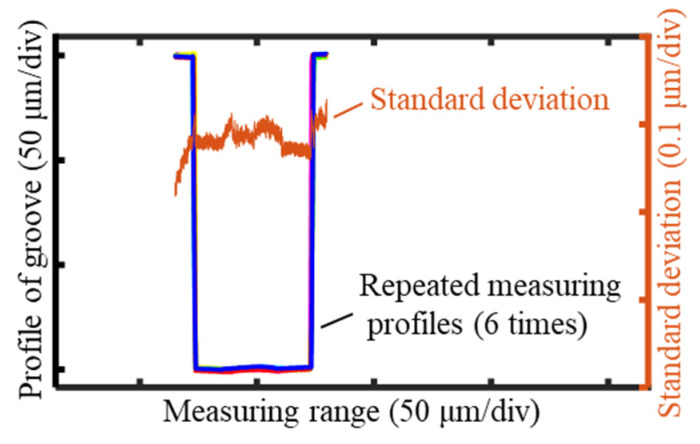
Six repeated measuring results and the standard deviation (std = 82 nm).

The standard deviation of the measurement results was used as the assessment criterion to evaluate the accuracy of the deflection-based measurement method and system. The measurement and profile-stitching experiments were repeated six times, and the results are presented in Figure 24. The standard deviation of all measurement points from the six repeated experiments was calculated to assess the precision of the entire measurement process. The computed standard deviation of the full cross-sectional groove profile was 82 nm, indicating the system’s high measurement accuracy.

The standard deviation progressively increased from left to right along the groove profile. During the profile stitching process, the left-side profile was fixed as the reference, while the right-side profile was adjusted for alignment. As a result, the measurement repeatability of the left-side profile was higher. The stitching region was located at the bottom of the profile, where the point cloud alignment introduced errors, which prevented a perfect overlap of all the measurement points. The nearly 90° angle between the groove’s sidewall and the bottom also amplified the stitching error. This amplification propagated through the angle, which led to a gradual decline in the measurement repeatability along the height of the right-side profile. As a result, the standard deviation increased progressively with the height.

The measured standard deviation of 82 nm included the effective profiles of the groove’s sidewalls and bottom and the planar region at the top of the groove. When evaluating the measurement accuracy of the groove profile, the planar region at the top of the groove could be excluded, with a sole focus on the profiles of the sidewalls and bottom. The standard deviation increased progressively from the left to the right side of the groove, where the planar region at the top of the right side exhibited the highest standard deviation. Therefore, by excluding the planar region at the top, which was not required for assessing machining accuracy, the repeatability of the measurement system was found to be better than 82 nm.

The factors that influenced the measurement uncertainty included the probe resolution, laser sensor accuracy, capacitive sensor accuracy, and the repetitive positioning accuracy of the Z-axis. The resolution of the probe was determined by its diameter. A custom-developed large length-to-diameter ratio probe was utilized, which featured a resolution of 20 nm, as illustrated in Figure 12. The laser and capacitive sensors’ uncertainty was influenced by the sensor resolution, temperature drift, and environmental noise. Considering these factors, the measurement uncertainty of the laser sensor was determined to be 1 nm, while that of the capacitive sensor was 3 nm. The coordinate output of the Z-axis was used to provide feedback on the profile information of the groove, with a repetitive positioning accuracy of 50 nm within the measurement range.

The measurement system also included the X-axis motion module. Since the straightness error of the X-axis was separated during the measurement process using the capacitive sensor, its straightness error was determined by the sensor’s measurement accuracy and was not considered separately. Environmental factors, such as the temperature and humidity, influenced the system’s uncertainty by reducing the accuracy of the sensors. These effects were accounted for by incorporating the evaluated measurement uncertainties of the sensors.

The factors that influenced the uncertainty of the measurement system are summarized in Table 3, and the uncertainty was evaluated using the Guide to the Expression of Uncertainty in Measurement (GUM) method [39]. The calculation formula is presented as follows:

(17) $$u_{system}=\sqrt{{u_{probe}}^{2}+{u_{laser}}^{2}+{u_{capacitive}}^{2}+{u_{Z-axis}}^{2}}$$

According to Equation (17), the uncertainty of the measurement system was determined to be 54 nm. The expanded uncertainty was calculated to be 108 nm, with a coverage factor of 2, which corresponded to a 95% confidence interval for the measurement results.

The repeatability and uncertainty analysis of the measurement system confirmed its accuracy at approximately the 100 nm level. The system effectively captured the profile information of the high-aspect-ratio micro-groove, which demonstrated the reliability and practicality of both the measurement system and the deflection-based measurement method.

## 5. Conclusions

A novel measurement method for the full cross-sectional profile for high-aspect-ratio micro-grooves was proposed based on deflection probes. A deflection-based scanning measurement system was established that enables the acquisition of the complete cross-sectional profile of high-aspect-ratio micro-grooves by stitching the positive and reversal deflection-based measurement results. The method effectively eliminated measurement blind areas at the groove bottom and sidewalls.

A large length-to-diameter ratio probe was developed to scan the profile of high-aspect-ratio micro-groove. During the measurement process, a force feedback structure maintained the contact force between the probe and the workpiece at 0.48 mN. Considering profile stitching and minimizing the probe deformation, the optimal deflection angle for the deflection-based measurement method was determined to be 8.5°. Two CCD sensors were used to calibrate the relative position error of the probe and groove before each measurement. Integrating a laser sensor and a reflector into the measurement system effectively separated the straightness error of the X-axis from the measurement results. The complete cross-sectional profile of the groove was reconstructed by stitching the overlapping region at the groove bottom through the point cloud registration method, which verified that the measurement method could achieve a micro-groove measurement with a width of 50 μm and an aspect ratio of not less than 3.

The full-process groove measurement experiment was repeated six times, which resulted in a standard deviation of 82 nm for the measurement results. The sources of the measurement errors were analyzed, and the expanded uncertainty of the measurement results was calculated to be 108 nm. The experimental results demonstrated the capacity of the proposed measurement system for measuring the full cross-sectional profile of high-aspect-ratio micro-grooves with nano-precision.

## Figures and Tables

**Table 1:** Specifications of the measuring device.

	Items	Values
X/Z-axes	Measuring range	150 mm
	Repetitive positioning accuracy	50 nm
Capacitive sensor	Resolution	3 nm
Probe	Tip diameter	18 nm
	Length–diameter ratio	10:1
Laser sensor	Measuring range	2.1 mm
	Resolution	1 nm

**Table 2:** The parameters of the measured groove profile.

	Symbol	Left Side	Right Side
Width	*d* (μm)	50.7	
Depth	*h* (μm)	149.23	150.43
Angle	*θ* (°)	90.3	89.7
Roughness	*Ra* (nm)	55	58

**Table 3:** Uncertainty budget of the measuring system.

Uncertainty Sources	Type	Symbol	Standard Uncertainty (nm)
Uncertainty of measuring system	B	*u*_system_	54
Resolution of probe	B	*u*_probe_	20
Accuracy of laser sensor	B	*u*_laser_	1
Accuracy of capacitive sensor	B	*u*_capacitive_	3
Repetitive positioning accuracy of Z-axis	B	*u*_z-axis_	50

## Data Availability

The data underlying the results presented in this paper are available from the authors upon request.

## Abbreviations

The following abbreviation is used in this manuscript:

GUM	Guide to the Expression of Uncertainty in Measurement
